# *In‐situ* microbially induced Ca^2+^‐alginate polymeric sealant for seepage control in porous materials

**DOI:** 10.1111/1751-7915.13315

**Published:** 2018-10-06

**Authors:** Liang Cheng, Yang Yang, Jian Chu

**Affiliations:** ^1^ Department of Civil and Environmental Engineering Nanyang Technological University 50 Nanyang Avenue Singapore City 639798 Singapore

## Abstract

This paper presents a novel approach of using *in‐situ* microbially induced Ca^2+^‐alginate polymeric sealant for seepage control in porous materials. This process comprises two steps: (i) generation of insoluble calcium carbonate inside the pores of porous materials (such as sand) through a microbially induced carbonate precipitation (MICP) process *in‐situ* and (ii) injection of sodium alginate for *in‐situ* gelation via reaction between alginate and Ca^2+^ ions. The experimental results showed that the hydraulic conductivity/permeability of sand decreased with the increase in alginate concentration. When 5% alginate was used with a CaCO_3_ concentration of 0.18 g g^−1^ sand, the permeability of the alginate‐treated sand reduced from 5.0 × 10^−4^ to 2.2 × 10^−9^ m s^−1^. The scanning electron microscopy images revealed that a film‐type coating was formed around sand particles with spherical round crystals embedded. Furthermore, the *in‐situ* formed Ca‐alginate polymeric sealant can also be used for the removal of Cu^2+^ ion and suspended particles from contaminated water by more than 90%. Built on the current research, the envisioned practical application of the proposed method may include clogging fractured rock, reducing seepage and prevent piping through dams, excavation dewatering, and forming barriers for remediating specific contaminants.

## Introduction

It has been well recognized that seepage flow has a great influence on the stability of geotechnical structures and the safety of environment. Therefore, seepage control is a crucial design and construction process for many infrastructures such as reservoirs, earth dams, tunnels and other underground constructions. Various solutions have been proposed for seepage control, e.g. cement grouting, chemical grouting, or slurry cut‐off walls (Xanthakos *et al*., 1994; Chu *et al*., 2009).

One common method for seepage control is to change the internal hydraulic properties of the materials, such as the permeability of sand by injection of sealant such as cement, chemicals or polymers (e.g. polyurethane prepolymer) into the pores of the porous materials to stop water seepage (Chen *et al*., 2010; Lisa *et al*., 2013; Lentz, 2015). However, the pores in some of the porous materials such as fine sand are too small for sealant to permeate. It is also difficult to inject viscous sealant such as cement or polymers into soil. Furthermore, there is also environmental concern related to the unrecoverable change of the natural soil due to the injection of cement or chemical grouts (Farah *et al*., 2016).

As alternatives to those conventional seepage control techniques, biological approaches are now being intensively studied. These include microbe injection and byproduct precipitation. To date, Microbially Induced Carbonate Precipitation (MICP) process based biogrout material has been developed and explored for various applications in geotechnical engineering. Clogging of soil through MICP has several advantages over the conventional methods as the biogrout material has low viscosity in solution thus can penetrate better than cement or chemical grouts (Chu *et al*., 2014) and more environmental friendly (DeJong *et al*., 2010). However, the MICP approach also has its shortcomings. One of the major concerns is the generation of highly concentrated byproduct of ammonium chloride potentially harmful to environment, especially groundwater. Another technique is to use microbially produced biofilms and extracellular polymeric substance (EPS) in the pores of soils (Baveye *et al*., 1998). However, the durability of biofilms and EPS over time is a concern. Furthermore, this approach could be relatively slow to achieve a significant permeability reduction of soils as it requires the formation of a bulk biomass and biofilm at the macroscopic level (Seifert and Engesgaard, 2007; Zhong and Wu, 2012).

Instead of cultivating the microorganisms and biofilm in the soil, another approach is to use biogenic excrement (i.e. biopolymers), especially polysaccharides (e.g. alginic acid, alginate, xanthan, gum karaya, gum arabic) directly in soil to gain improved mechanical performance (Chang *et al*., 2016b). Low viscous polysaccharides by themselves normally cannot significantly reduce the hydraulic conductivity of porous materials unless the polysaccharide gelation can proceed to produce solid polymeric gel that can fill in the pores firmly. Usually, polysaccharides form gel when the polymeric carbohydrate molecules react with soluble divalent metal ions, leading to rapid crosslinking reaction and instant solid gel formation (Lee and Mooney, 2012). This rapid and poorly controllable gelation process is not applicable for large scale field application. This is because it is difficult, if not impossible, to inject low viscous polysaccharide solution over a long distance before it turns into solid gel form.

To overcome the aforementioned disadvantages, the current study presents a new method for soil hydraulic conductivity reduction and seepage control by injection of low viscous alginate solution (a type of polysaccharide) followed by an *in‐situ* slow and controllable gelation process. This slow gelation process is achieved by gradual formation of Ca‐alginate gel inside the soil via a novel process of *in‐situ* bio‐acidification and bio‐leaching of Ca^2+^ ions from insoluble CaCO_3_ crystals which are produced through microbially induced carbonate precipitation (MICP). This new method makes it possible to control seepage in porous materials with small pore sizes such as fine sand through injection method.

To develop an effective method, several parameters related to the formation of microbially induced Ca^2+^‐alginate polymeric sealant inside the sand were examined and discussed. These included alginate concentration, gelation time, long‐term stability, uniformity, and heavy metal and suspended particles removal. This study has clearly demonstrated that it is feasible to use this method for seepage control in soils and copper ion removal. Thus, the proposed method could potentially provide solutions to many geotechnical and environmental problems such as seepage control for underground construction and remediation of specific contaminants.

## Principle of process

**Figure 1:**
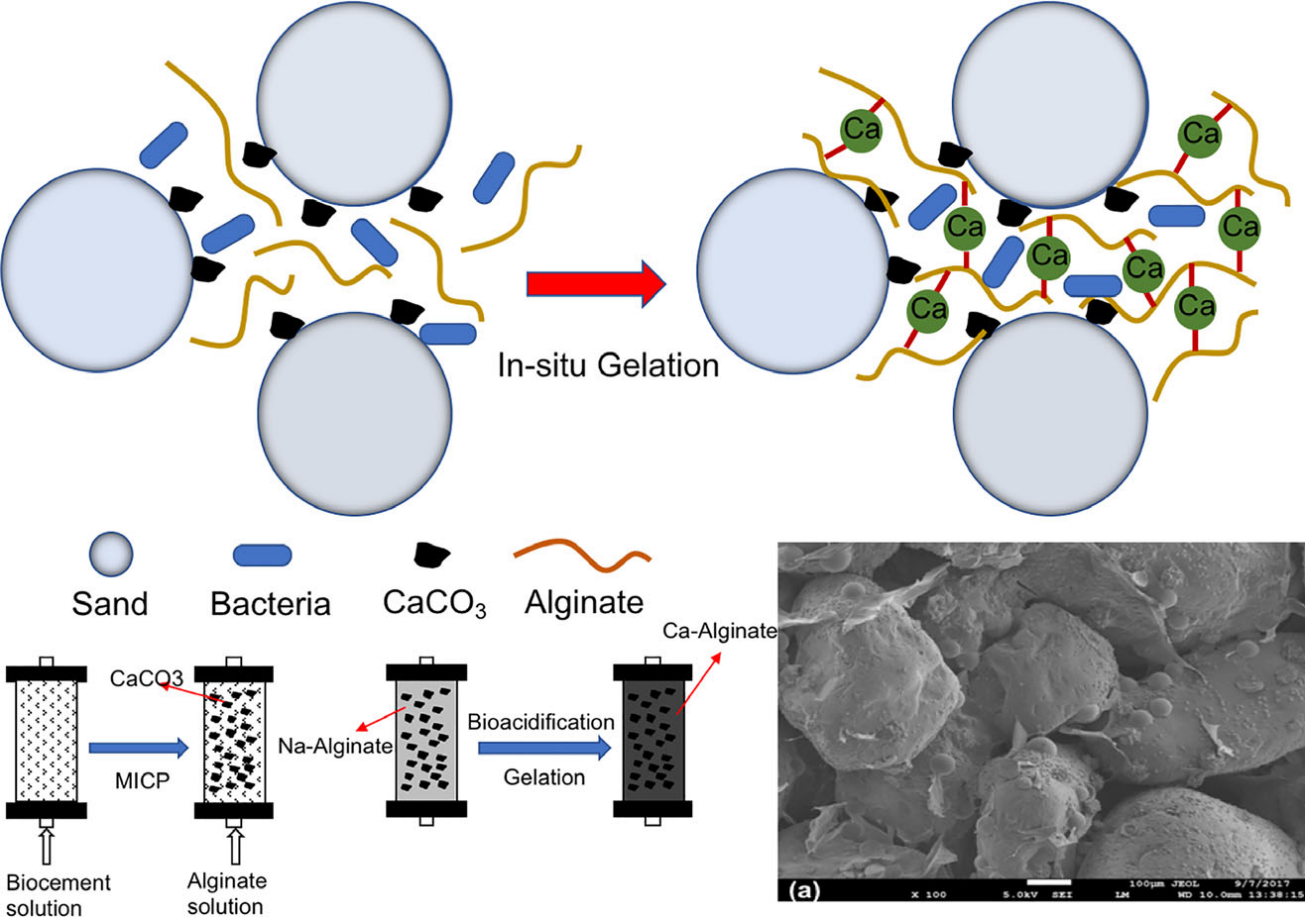
Illustration of *in‐situ* microbially induced Ca^2+^‐alginate polymeric sealant and treatment procedure.

The *in‐situ* gelation is achieved when the injected alginate polymer reacts with the slowly dissociated calcium ions as a result of CaCO_3_ dissolution via anaerobic fermentation process. The overall process was illustrated in Fig. 1. The principle of *in‐situ* alginate gelation can be described as follows:

Step 1: *In‐situ* generation of CaCO_3_ at the pores of soil through MICP (Cheng *et al*., 2013) (Eq. (1)):

(1) $$\begin{array}{cc} & \text{CO}{\left({\text{NH}}_{2}\right)}_{2}\left(\text{urea}\right)+2H_{2}O\\ & +{\text{Ca}}^{2+}\overset{\text{Ureolytic Bacteria}}{\to }{\text{CaCO}}_{3}↓+2{\text{NH}}_{4}^{+} \end{array}$$

Step 2: Gelation of Ca‐alginate which takes place after the injection of alginate solution consisting of sodium alginate, microorganisms and organic carbon (i.e. glucose) into the soil.

The following biochemical reactions are involved:

1. In the presence of organic carbon, the alginate solution turns to acid due to the bio‐acidification as a result of anaerobic fermentation. An example of bio‐acidification reaction via anaerobic fermentation is given as follows (Eq. (2)):
  (2) $$\begin{array}{cc} & C_{6}H_{12}O_{6}+2{\text{NAD}}^{+}+2\text{ADP}\\ & +2P_{i}\to 2{\text{CH}}_{3}{\text{COCOO}}^{-}+2\text{NADH}+2\text{ATP}+2H_{2}O+2H^{+} \end{array}$$
2. The produced protons (H^+^) react with the CaCO_3_ to produce soluble Ca^2+^ cations. An example of Ca ions dissociation is given as follows (Eq. (3)):
  (3) $$\begin{array}{c} 2H{}^{+}+{\text{CaCO}}_{3}\to {\text{Ca}}^{2+}2{\text{HCO}}_{3}^{2}- \end{array}$$
3. The dissociated Ca^2+^ ions react with alginate to form gel, as indicated as follows (Eq. (4)):
  (4) $$\begin{array}{cc} & {\text{Ca}}^{2+}+C_{6}H_{9}{\text{Na}\;}_{7}\left(\text{Sodium alginate}\right)\to \\ & {\left(C_{12}H_{14}{\text{CaO}}_{12}\right)}_{m}\left(\text{Ca}\text{‐}\text{Alginate}\;\left(\text{gel}\right)\right)+{\text{Na}}^{+} \end{array}$$

## Results

### Bio‐acidification enables Ca^2+^ dissociation from CaCO_3_

**Figure 2:**
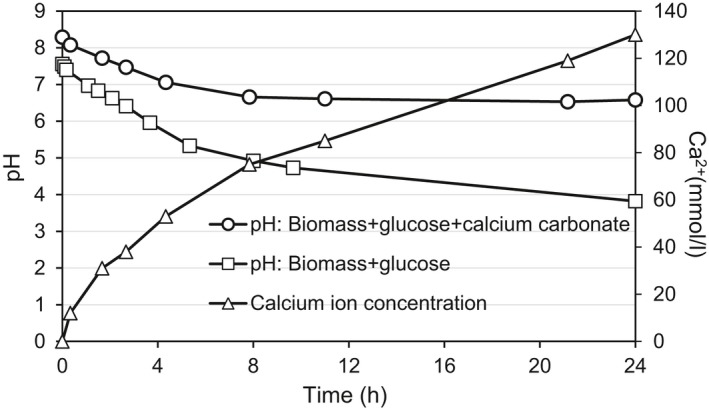
Effect of bio‐acidification on pH evolution and Ca^2+^ leaching from calcium carbonate crystals.

In this study, the process of CaCO_3_ dissolution based on the aforementioned bio‐acidification process was first investigated in bulk solution. By mixing the cultivated mixed bacterial culture with the carbon source of glucose, the pH of the system continuously decreased throughout the experiment to about 3.5 after 24 h of anaerobic fermentation. This suggested a continuous production of proton (H^+^), which could lead to a dissociation of Ca^2+^ from the CaCO_3_ crystals as shown in Fig. 2. The production of soluble Ca^2+^ increased over time to about 120 mM after 24 h. This Ca^2+^ concentration was adequate to proceed the gelation of alginate (Mahamadi and Zambara, 2013).

### Effect of incubation period on seepage rate

**Figure 3:**
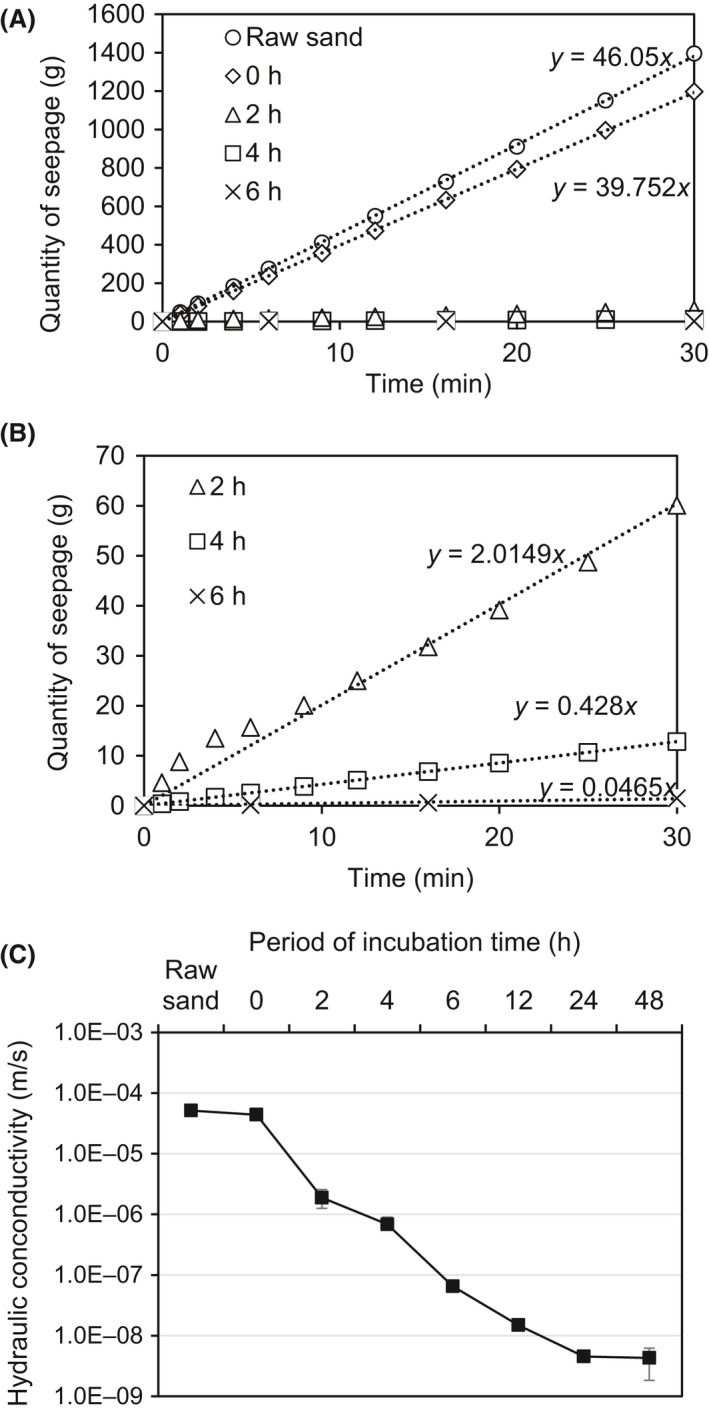
The evolution of (A and B) seepage rate and (C) hydraulic conductivity of sand columns treated with 5 g l^−1^ Na‐alginate gel associated with slowly produced Ca^2+^ dissociated from the MICP produced CaCO_3_ via bio‐acidification.

The above test clearly showed a gradual decrease in solution pH and increase in soluble Ca^2+^ ion concentration in aqueous phase. Therefore, it is well known that the gradually leached Ca^2+^ ions would result in a slow gelation process, thus a development of seepage reduction. By investigating the evolution of the seepage rate (hydraulic gradient of 1 m) after the introduction of the alginate solution, it was found that the initial seepage rate of the sand column treated with MICP only was about 46 ml min^−1^, which was then slightly reduced to about 39.7 ml min^−1^ after the introduction of alginate solution (Fig. 3A). A rapid decrease in the seepage rate was obtained after 2 h of incubation, which was further decreased dramatically with the increase in incubation time. For example, after 2 h of incubation at room temperature, the seepage rate was reduced by 95% to about 2 ml min^−1^ (Fig. 3B). After 12 h of incubation, the seepage rate was not detectable within the measuring period of 30 min. The hydraulic conductivity of the sand columns was also calculated, which decreased with the increase in the incubation time up to 24 h. It was found that the hydraulic conductivity reduced from 4.4 × 10^−5^ to 4.5 × 10^−9^ m s^−1^, which was similar to well‐compacted kaolin clay (Boynton and Daniel, 1985) (Fig. 3C).

### Effect of alginate concentration on hydraulic conductivity reduction

**Figure 4:**
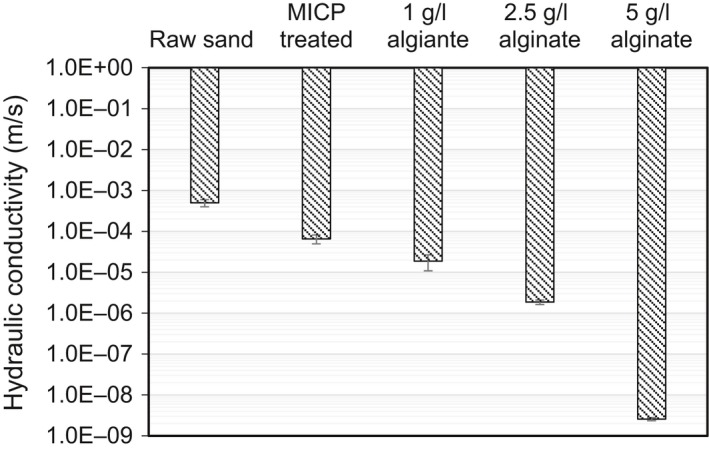
Changes in hydraulic conductivity as function of alginate concentration (24 h of incubation).

The hydraulic conductivity reduction of the alginate treated soils was depended on the alginate concentration used, which was decreased from 6.56 × 10^−5^ to 1.87 × 10^−5^, 1.86 × 10^−6^, and 2.58 × 10^−9^ m s^−1^ with 1, 2.5 and 5 g l^−1^ alginate used respectively (Fig. 4). Usually, compressive modulus and strength of Ca‐alginate gel increased with polymer concentration, which is attributed to the increase in polymer chain density and entanglement (Kuo and Ma, 2001). The higher concentration of Ca‐alginate gel with enhanced mechanical properties might allow more firm attachment of gel on the sand grain surface, preventing it from being flushed out. Thus, it leads to completed pore blocking with denser three‐dimensional cross‐linked network and results in lower hydraulic conductivity. It should be noted that for the current sand column alginate concentration higher than 5 g l^−1^ alginate (i.e. 7.5 g l^−1^) would lead to severe surface clogging and failure of treating the entire sand column, which might be due to the high viscosity of the alginate solution (data not shown).

### Uniformity and durability

**Figure 5:**
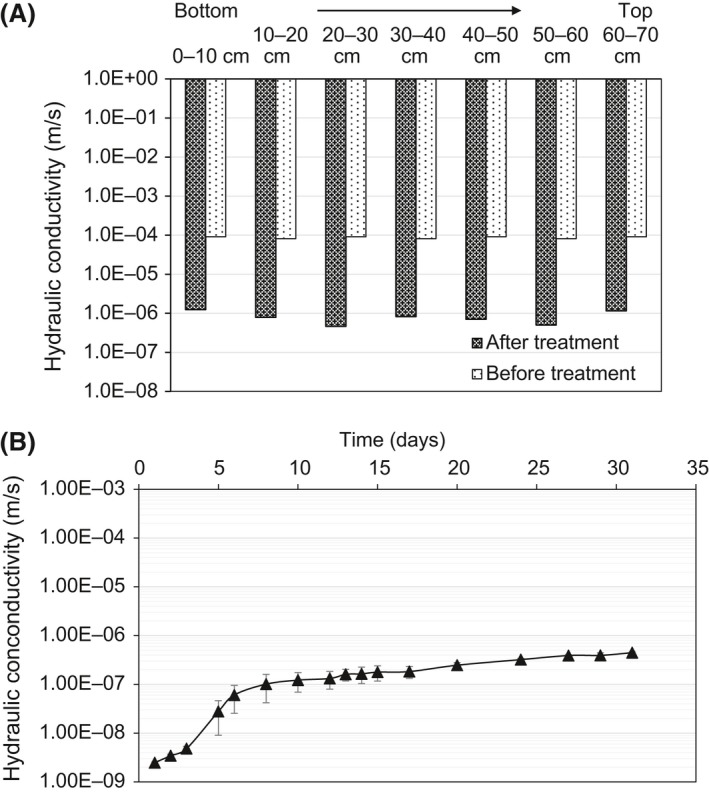
(A) Uniformity and (B) durability of sand column treated with microbially induced Ca^2+^‐alginate gel.

The hydraulic conduction of different sections of a long sand column clogged by the *in‐situ* formed Ca‐alginate gel is shown in Fig. 5A. The result shows that for each section the hydraulic conductivity reduction is very substantial (more than 2 magnitudes) and relatively homogeneous, from 8.69 ± 1.31 × 10^−5^ m s^−1^ before the gelation to 8.14 ± 2.12 × 10^−7^ m s^−1^ after the gelation (24 h incubation). Comparing with the bioclogging driven by proliferation of bacteria and formation of bacteria colonies at the pore spaces in the presence of continuous supply of nutrients done by previous research, the current *in‐situ* formed Ca‐alginate gel polymer resulted in a more uniform clogging and hydraulic conductivity reduction (Zhong and Wu, 2012).

The durability of the *in‐situ* formed alginate gel was evaluated by measuring the hydraulic conductivity as a function of time. The results showed that the initial hydraulic conductivity of the sand column was about 2.49 × 10^−9^ m s^−1^. After 5 days, the hydraulic conductivity increased to 2.78 × 10^−8^ m s^−1^. After 1 month, the hydraulic conductivity of the column was slowly increased to around 4.47 × 10^−7^ m s^−1^ (Fig. 5B). This increase in the hydraulic conductivity could be due to the degradation of the Ca‐alginate. It has been reported that the alginate gel formed using ionically cross‐link approach (i.e. using Ca^2+^ to cross‐link alginate) can be slowly dissolved again because of the release of divalent ions due to exchange reactions with monovalent cations (Lee and Mooney, 2012). The final value of 4.47 × 10^−7^ m s^−1^ was still 10 times lower than that of the sand column clogged by bacteria augmentation and biofilm formation after a long‐term treatment (e.g. months) (Kanmani *et al*., 2014). The alginate treated sand with low hydraulic conductivity can be used as biopolymer barrier for temporary waterproof engineering applications. Further retrieving of the low hydraulic conductivity by repeated treatment could be a solution for a long‐term seepage control. However, for some specific geotechnical applications long‐term (i.e. months) stability may be needed, which requires further study in the future.

### Microstructure analysis

**Figure 6:**
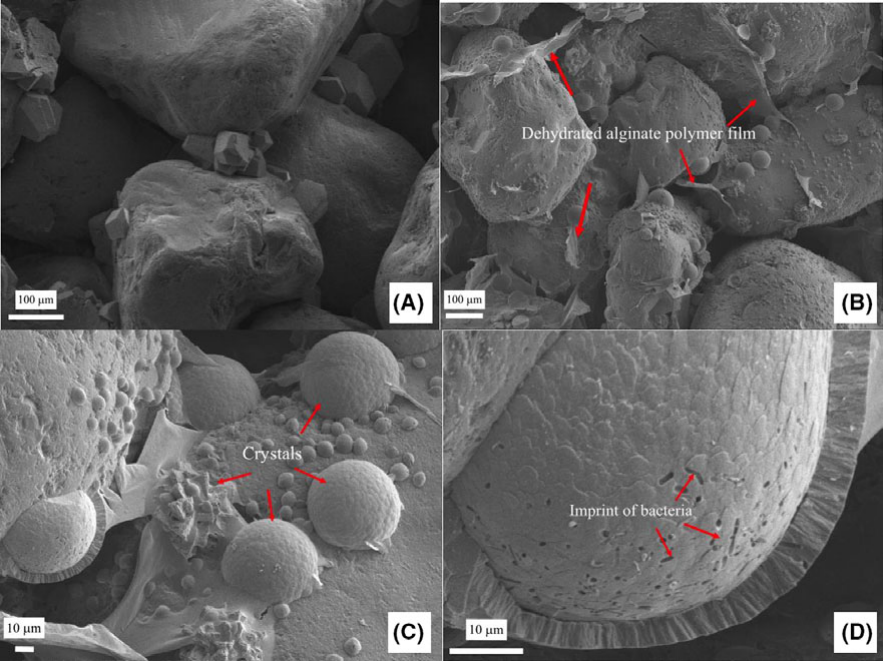
Microstructure of MICP (A) and Na‐alginate treated sand samples after gelation: (B) the sand particles connected with alginate polymer and crystal precipitates; (C) gel polymer and spherical crystals formed between sand particles; (D) spherical crystal with imprint of bacterial cells on the surface. The dehydrated alginate gel polymer film, crystals and imprint of bacteria are indicated by the red arrows in the images (B), (C) and (D).

The microstructure SEM analysis was conducted on freeze‐dry sand samples after the *in‐situ* gelation of Ca‐alginate. Figure 6 shows representative SEM images of sands collected from the experimental columns. Figure 6A indicates the sand grains collected from the sand column treated by MICP only were connected by rhombohedral shape MICP induced CaCO_3_ crystals, which are in line with many previous studies (Cheng and Cord‐Ruwisch, 2012; Cheng *et al*., 2017). Figure 6B and C show sand grains connected or covered by a film‐type gelatinous material believed to be the Ca‐alginate. This is because the sample preparation (i.e. drying process) prior to the SEM measurement would lead to a dehydration of hydrogel, resulting in a film‐type structure shown in the SEM images (Chang *et al*., 2016a). The typical rhombohedral shape CaCO_3_ crystals are not observed in the samples collected from the sand columns treated with alginate solution. Spherical shape of CaCO_3_ crystals with imprints of the bacterial cells at the surface was clearly observed (Fig. 6D). The transfer of rhombohedral to spherical shape crystals might be due to the CaCO_3_ dissolution and recrystallization in the presence of alginate polymer, which has been proved to result in dramatic changes in the appearance of the crystals due to the direct adsorption of the alginate on the crystal surface (Lakshtanov *et al*., 2015).

### Cu^2+^ removal and turbidity reduction

**Figure 7:**
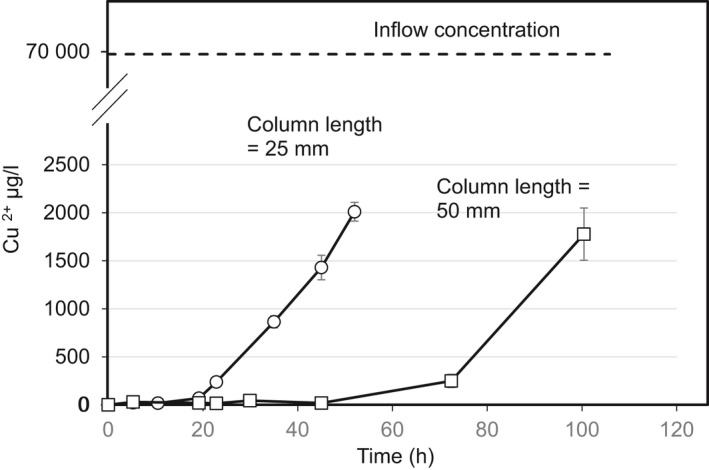
Cu^2+^ ion concentration of the effluent obtained from Ca‐alginate clogged sand columns.

The Cu^2+^ ions adsorption when passing through the alginate treated sand columns (different lengths of 25 and 50 mm) at an average seepage rate of 2 ml h^−1^ is illustrated in Fig. 7. The results show that the curve follows the typical characteristic profile produced in ideal Cu adsorption systems (Mahamadi and Zambara, 2013). The time when a noticeable Cu detected in the effluent seepage varied with column length with a displacement of the front of adsorption being observed at higher length. When the column length was reduced, axial dispersion phenomena predominated in the mass transfer and reduced the diffusion of metallic ions (Mahamadi and Zambara, 2013), suggesting that the solute (metallic ion) had not enough contact time (constant flow through a shorter column) to diffuse into the whole of the adsorbent mass (Taty‐Costodes *et al*., 2005). Also, the longer sand columns filled with greater amount of Ca‐alginate gel would provide more binding sites for adsorption (Baral *et al*., 2008).

The Ca‐alginate clogged sand columns were also tested for turbidity reduction. The total volume of the liquid passed through the sand columns was about 10 void volume. The initial turbidity of the supplied influents was stable throughout the experiment. The changes in the turbidity are shown in Table 1. Overall, the amount of particulate matter removal determined from the turbidity measurement, including inorganic material of clay and organic material of bacteria, was more than 90% much higher than that obtained from control columns. The highly efficient particulate matter filtration could be due to the smaller pore sizes after occupied by the *in‐situ* formed Ca‐alginate gel and particles entrapment by the alginate polymer. It is well known that extracellular polymeric substances can serve to trap and store particulates (Rinck‐Pfeiffer, 2000).

**Table 1:** Turbidity changes during water passage through sand columns with *in‐situ* formed Ca‐alginate gel

Water type	Inflow (NTU)	Outflow (NTU)	% Removal	Column
RW	27.6 ± 0.23	2.1 ± 0.07	92.4 ± 0.2	Ca‐alginate
		16.0 ± 0.95	41.9 ± 3.9	Control
WB	33.3 ± 0.42	3.2 ± 0.11	90.4 ± 0.2	Ca‐alginate
		28.5 ± 1.06	14.4 ± 2.1	Control
WC	124 ± 2	6.81 ± 0.45	94.5 ± 0.3	Ca‐alginate
		37.4 ± 3.1	69.6 ± 2.0	Control

RW, real wastewater after primary treatment; WB, deionised water with 9% NaCl and *Escherichia coli*; WC, deionised water with kaolin clay.

## Discussion

### *In‐situ* bio‐leached Ca^2+^ enables gradual polymeric gelation

In the current study, an anaerobic fermentation in a MICP pretreated sand column was carried out to induce *in‐situ* bio‐leaching of Ca^2+^ that could be further utilized for polymeric gelation of alginate. Bio‐acidification and bio‐leaching are common microbiological processes that are widely employed in many different engineering applications, such as metals extraction from ores. Bio‐acidification due to an anaerobically biological production of lactic acid has been reported (Fan *et al*., 2017). In the absence of oxygen as usually encountered in a subsoil environment, microorganisms such as fermentative anaerobic organisms use the lactic acid fermentation pathway to gain energy. This type of metabolism results in a natural bio‐acidification process leading to mobilization of metals (Xie *et al*., 2015), such as Ca^2+^ ions in the current study. The *in‐situ* acidity would dissolve calcite producing free calcium ions and releasing carbon dioxide which would in‐turn enhances the growth of anaerobic bacteria.

CaCl_2_ is one of the most frequently used agents to initiate the cross‐linking of alginate; however, it typically leads to rapid and poorly controlled gelation due to its high solubility in aqueous solutions (Lee and Mooney, 2012). To slow down and control the gelation rate, the current applied approach utilizes bio‐acidification pathway to dissociate Ca^2+^ from the CaCO_3_ by lowering the pH gradually *in‐situ*. The released Ca^2+^ subsequently induces the gelation of the alginate solution in a more gradual way. Therefore, the biopolymer of Ca‐alginate clogging did not happen immediately after the injection of the alginate solution and a certain period of incubation was required to enable an effective seepage control.

### Gradual gelation enables uniform permeability reduction

Comparing with chemical reaction (i.e. CaCl_2_ reacts with alginate) which is a rather straightforward process, the microbial metabolism related bio‐acidification and bio‐leaching reactions are much slower, resulting in a gradual gelation process (slow gelation rate) (Fig. 3C). The current system provides a new method of reducing the gelation rate, which is favourable to widen the working time for alginate gels and beneficial for large scale field application. The release of protons from the glucose anaerobic fermentation gradually liberated calcium ions from the insoluble CaCO_3_ in an acid‐based reaction. As the association of the polyguluronate sequences of the alginate was triggered by the progressively produced calcium ions, alginate clusters expanded in size until forming a continuous uniform three‐dimensional crosslinked network (Farrés and Norton, 2014), which increased the contribution of hydraulic conductivity reduction. Furthermore, the gelation rate is believed to be a critical factor in controlling gel uniformity and strength when using divalent cations (Lee and Mooney, 2012). Kuo and Ma (2001) found that slower gelation produces more uniform structure and greater mechanical integrity.

The slow gelation process also produced a uniform distribution of the gelated alginate filling out the pores between sand particles at macro‐scale. This results in a more uniform permeability reduction compared with the permeability reduction driven by biofilm clogging (Fig. 5A). This is because for the biofilm clogging the conditions (e.g. nutrient concentrations, dissolved oxygen concentration, etc.) that can enhance the biofilm growth always varies along the sand column (Zhong *et al*., 2013; Farah *et al*., 2016), leading to an uneven proliferation of biofilm thus different levels of clogging and hydraulic conductivity reduction. In comparison, the current proposed approach is fundamentally driven by the chemical reaction between the slowly dissociated calcium ions and alginate polymeric molecules. The gelation induced by the slow production of Ca^2+^ would minimize the formation of inhomogeneous gelled networks due to the uniform distribution of Ca^2+^ ions throughout the system (Farrés and Norton, 2014).

### Possible practical applications

This study has demonstrated the strong promise of Ca‐alginate hydrogel for hydraulic permeability reduction. The use of microbially induced Ca‐alginate hydrogel for soil hydraulic conductivity control shows promising results to become an environmentally friendly alternative in *in‐situ* grouting purposes. However, it is worthwhile mentioning that the currently proposed method is not applicable for the porous materials which have the pore size smaller than bacteria. This is because the successful introduction of bacteria into the porous materials is essential for the first step of MICP and the second step of microbially induced gelation of alginate solution. With further development envisioned practical applications may include: clogging fractured rock, reducing seepage and prevent piping through dams, and temporary applications such as excavation dewatering (Proto *et al*., 2016).

As Ca‐alginate hydrogel is formed in the soil matrix, possible practical application of the proposed method may also be forming barriers for remediating specific contaminants. Many published researches have shown successful heavy metals (i.e. Pb^2+^, Cu^2+^, Zn^2+^) removal by Ca‐alginate gel as alginate provides bindings sites for adsorption of heavy metals (Park and Chae, 2004; Mahamadi and Zambara, 2013). The current approach also offers a potential solution to turn a natural soil bed into a safety barrier for an efficient heavy metal removal. It is well known that flow rate is one of the important parameters that could affect the metal adsorption by alginate gel (Vinodhini and Das, 2010). The low hydraulic conductivity of the Ca‐alginate clogged sand column resulted in a low flow rate of the seepage and dramatically increased the contact time between the metal ions and the adsorbent, resulting in substantial metal ions adsorption and a high bed adsorption capacity (Taty‐Costodes *et al*., 2005). Therefore, the current invented technology would not only provide alternative approach for *in‐situ* seepage control, but also be able to offer a safety barrier that could entrap toxic chemicals (i.e. heavy metals) and prevent them from entering into underground water. Future study will be focused on competitive adsorptive removal of various types of heavy metals, i.e. Pb^2+^, Cu^2+^, Cd^2+^, Zn^2+^, etc. and their effects on the MICP process and Ca‐alginate formation.

The soil zone treated with the *in‐situ* formed Ca‐alginate gel could be a promising method to control the seepage of contaminants to the surrounding environment and manage the contaminants migration in the underground space. As a promising remedial manner, further systematic evaluation on the current approach for heavy metals and organic contaminants removal will be carried out using synthetic and/or real contaminated solution.

As microbe metabolism and enzyme activity were involved in the proposed method, the environmental temperature would have a significant effect on the efficiency of the process. It has been shown that for very low temperature (i.e. 4°C), the MICP process would be of much lower efficiency compared with the room temperature of about 25°C (Cheng *et al*., 2017). Therefore, the environmental temperature needs to be seriously taken into account when applying the proposed in real situations.

## Conclusions

In this paper, a new approach of using *in‐situ* microbially induced Ca^2+^‐alginate polymeric sealant for seepage control of porous materials was presented. The slow release of Ca^2+^ ions from the CaCO_3_ produced *in‐situ* through the MICP process resulted in a gradual gelation and uniform distribution of Ca‐alginate gel. The ability to control the gel time is one of the major advantages of this method as this is particularly beneficial for large scale field applications. The laboratory experimental results show that the hydraulic conductivity decreased with the increase in alginate concentration. For 5% alginate used, the hydraulic conductivity of the alginate treated sand reduced from 5 × 10^−4^ to 2.2 × 10^−9^ m s^−1^. The sand bed filled with the *in‐situ* formed Ca‐alginate polymeric sealant also shows efficient removal of Cu^2+^ ions as well as suspended particles by more than 90%. This suggests that in addition to seepage control, this method could also be used for remediation of specific contaminants by entrapping the toxic chemicals (i.e. heavy metals) and preventing them from leaking into surrounding environment.

## Experimental procedures

### Sand and chemicals

Ottawa sand was used with the following grain size distribution: > 1.18 mm 0%; 0.6–1.18 mm 3.22%; 0.6–0.425 mm 26.05%; 0.3–0.425 mm 43.03%; 0.15–0.3 mm 27.08%; < 0.15 mm 0.62%. All chemicals are commercially available and analytical grade used. Yeast extract (YE), calcium chloride, urea, potassium dihydrogen phosphate, nickel chloride, d‐glucose, sodium alginate, sodium hydroxide, hydrochloric acid and CuSO_4_·5H_2_O were purchased from Sigma‐Aldrich (Singapore). Deionized water was used in all experiments.

### Microorganisms

The urease active microorganism used in current study for driving MICP process was *Bacillus* sp. Isolated from activated sludge using the similar method as indicated by Al‐Thawadi and Cord‐Ruwisch (Al‐Thawadi and Cord‐Ruwisch, 2012). The microorganism was cultivated using the recipes indicated in the previous study (Cheng and Shahin, 2016). The microorganism was grown to early stationary phase (24 h to 48 h incubation at 28°C) and stored at 4°C (no longer than 2 weeks) prior to use. The collected culture had optical density (OD_600_) about 4.2 ± 0.2 and urease activity about 20 ± 1 U ml^−1^ (1 *U* = 1 μmol urea hydrolysed per minute), which means the amount of urease enzyme contained in 1 ml of culture can hydrolyse 20 ± 1 μmol of urea per minute.

A mixed bacterial culture was utilized for bio‐acidification. The mixed culture was established with 10% activated sludge as inoculums, obtained from a local wastewater treatment plant (Singapore), and cultivated in YE‐based medium, containing YE (10 g l^−1^), KH_2_PO_4_ (2 g l^−1^) and pH = 7. After 24 h of incubation at 28°C, the culture was collected and centrifuged at a speed of 6000 r.p.m. for 10 min. The biomass was then resuspended in NaCl (9%) solution with optical density (OD_600_) of about 0.352 (30 times dilution) and stored at 4°C prior to use for the bio‐acidification process.

### Sample preparation

All columns used in this study for hydraulic conductivity measurement were made of polyvinyl chloride (PVC) tubing (internal diameter 50 mm, length 100 mm and 700 mm), and packed with Ottawa sand in six consecutive layers to achieve at least 90% of the maximum dry density of 1.67 g cm^−3^. The short sand columns were used for investing the parameters of incubation period, alginate concentration and durability. The long sand column was used for investing the parameter of uniformity. An inlet (bottom) of the sand column was connected to a peristaltic pump to allow an upward flow at a constant flow rate of about 1 l h^−1^. The effluent was collected from the top outlet. Scour pads and 10‐mm‐thick gravel were placed at the top and bottom of the column. For copper ions and suspended particles removal tests, the sand columns treated with 2.5% alginate solution samples were used.

### Treatment procedure

The proposed approach of using *in‐situ* microbially induced alginate‐Ca^2+^ polymeric sealant for permeability reduction of soils comprises two steps (Fig. 1). The first step is to introduce CaCO_3_ crystals into soils using MICP approach (using ureolytic bacteria to hydrolyse urea to produce CO_3_^2−^ ions which react with CaCl_2_ to produce CaCO_3_ crystals) following the procedure as stated in the previous study (Cheng *et al*., 2017). After the MICP process, the sand column was flushed with tap water to remove all the residual CaCl_2_. The average CaCO_3_ content was about 0.18 g g^−1^ sand by measuring the remained unreacted Ca^2+^ in the effluent. The second step is to introduce alginate solution (1.1 pore void volume), consisting of sodium alginate, the mixed microorganisms and d‐glucose, into the sand column until fully saturated. The alginate solution was injected from the bottom of the sand column using a peristaltic pump at a flow rate of about 1 l h^−1^. The alginate solution consisted of 5 g l^−1^ glucose, various concentration of sodium alginate ranging from 1–5 g l^−1^, 5% the mixed bacterial culture (v/v) and pH = 7.5 ± 0.1. After placing the sand column at room temperature for 24 h to allow the reaction of gelation to be completed, hydraulic conductivity test was conducted immediately. All experiments were conducted at room temperature (20 ± 2°C).

### Hydraulic conductivity measurement

The hydraulic conductivity test was conducted after the gelation process. Falling head permeability tests were conducted using the method described in AS 1289.6.7.2‐2001 (AS, 2001). Before conducting this test, water was flushed through the sample to remove any gas bubbles and achieve a steady flow.

### Microstructure analysis

After gelation, the sand sample was dried at 70°C for 48 h. Scanning electron microscopy (SEM) was conducted on the dried sand sample to characterize the structure and morphology of the *in‐situ* formed Ca^2+^‐alginate gel.

### Cu^2+^and suspended solid removal by column

Proto *et al*. (2016) have suggested that biopolymer formed within soil matrix may form barriers for remediating specific contaminants. In this study, in addition to the geotechnical application of seepage control, the potential application of remediation of contaminants by the microbially induced Ca‐alginate hydrogel was preliminarily evaluated by copper ion (Cu^2+^) and suspended solid removal. Copper contributes markedly to the environmental pollutions, especially of water and soil (Gibson and Mitchell, 2005), and it has been used as an example toxic heavy metal for pollution control (Kujan *et al*., 2005; Mahamadi and Zambara, 2013; Mahamadi *et al*., 2015). Different synthetic influents were used to evaluate the efficiency of Cu^2+^ and suspended solid removal by the Ca‐alginate treated sand columns (2.5 g l^−1^ alginate treated) under room temperature. Continuous flow experiments were conducted in the syringe columns to evaluate the efficiency of copper removal. The columns were operated in an up‐flow mode at room temperature with an average seepage rate of 2 ml h^−1^. The samples were collected from the effluent outlet at regular intervals of time and it was analysed for copper concentration using Inductively Coupled Plasma Mass Spectrometry (ICP‐MS). The suspended particles removal of three different types of solution, including real wastewater after primary treatment (RW), deionized (DI) water with 9% NaCl and *Escherichia coli* (WB), and DI water with kaolin clay (WC), was also conducted. Turbidity was measured using a HACH turbidity analyser and the sand columns without Ca‐alginate were also tested as control.

## Conflict of interest

None declared.
